# Literary Notes

**Published:** 1909-12

**Authors:** 


### LITERARY NOTES.

THE *American Journal of Surgery* announces that its December number will be a Philadelphia issue, the subject matter of which will consist entirely of contributions from among the leading men of that city. Among the subjects to appear and their contributors are as follows: A Consideration of the Diagnosis and Treatment of Retrodisplacement of the Uterus, E. E. Montgomery, Professor of Gynecology, Jefferson Medical College; Polypoid Growth of the Rectum and Report of a Recent Case, Lewis Adler, Jr., Professor of Diseases of the Rectum, Philadelphia Polyclinic; Tumors of the Urethra in Women, Barton Cooke Hirst, Professor of Obstetrics, University of Pennsylvania; The Control of Hemorrhage During Pregnancy, Edward P. Davis, Professor of Obstetrics, Jefferson Medical College; Cyclodialysis, Walter L. Pyle, Ophthalmologist to the Mt. Sinai Hospital, Assistant Surgeon of Willis Eye Hospital, Etc.; Röntgen Treatment of Malignant Diseases, Charles Lester

Leonard, Ex-President of the American Röntgen Ray Society; The Conservation of the Middle Turbinated Body, William A. Hitschler; The Diagnosis and Treatment of Ectopic Pregnancy, F. Brooke Bland.

The following well-known surgeons will also contribute and their titles will be announced at a later date. Ernest La Place, Professor of Surgery, Medical Chirurgical College; Professor William Campbell Posey, Professor of Ophthalmology, Philadelphia Polyclinic; John G. Clark, Professor of Gynecology, University of Pennsylvania; H. M. Christian, Clinical Professor of Genitourinary Diseases, Medical Chirurgical College; and John A. McGlinn.

